# Expression profiles of phytosulfokine signalling components in sorghum drought stress-adaptive response

**DOI:** 10.17912/micropub.biology.001284

**Published:** 2025-01-28

**Authors:** Tatenda Goche, Rudo Ngara, Stephen Chivasa

**Affiliations:** 1 Biosciences, Durham University, Durham, England, United Kingdom; 2 Crop Science, Bindura University of Science Education, Bindura, Zimbabwe; 3 Plant Sciences, University of the Free State, Bloemfontein, Free State, South Africa

## Abstract

Phytosulfokine (PSK) signalling promotes drought adaptation in drought-sensitive plants. However, whether naturally drought-tolerant plants deploy PSK signalling in drought is unknown. We are using two sorghum varieties with different drought response phenotypes to investigate tolerance mechanisms. We show that PSK signalling components have high constitutive expression before stress in the drought-tolerant variety. In contrast, gene expression is low in the drought-sensitive variety and is induced after drought exposure. Ability of the drought-tolerant sorghum variety to maintain elevated PSK signalling under optimal water availability suggests that genetic and physiological factors driving drought tolerance may be linked to elevated constitutive PSK signalling.

**Figure 1. Expression of the PSK gene network and drought-marker gene in sorghum.**
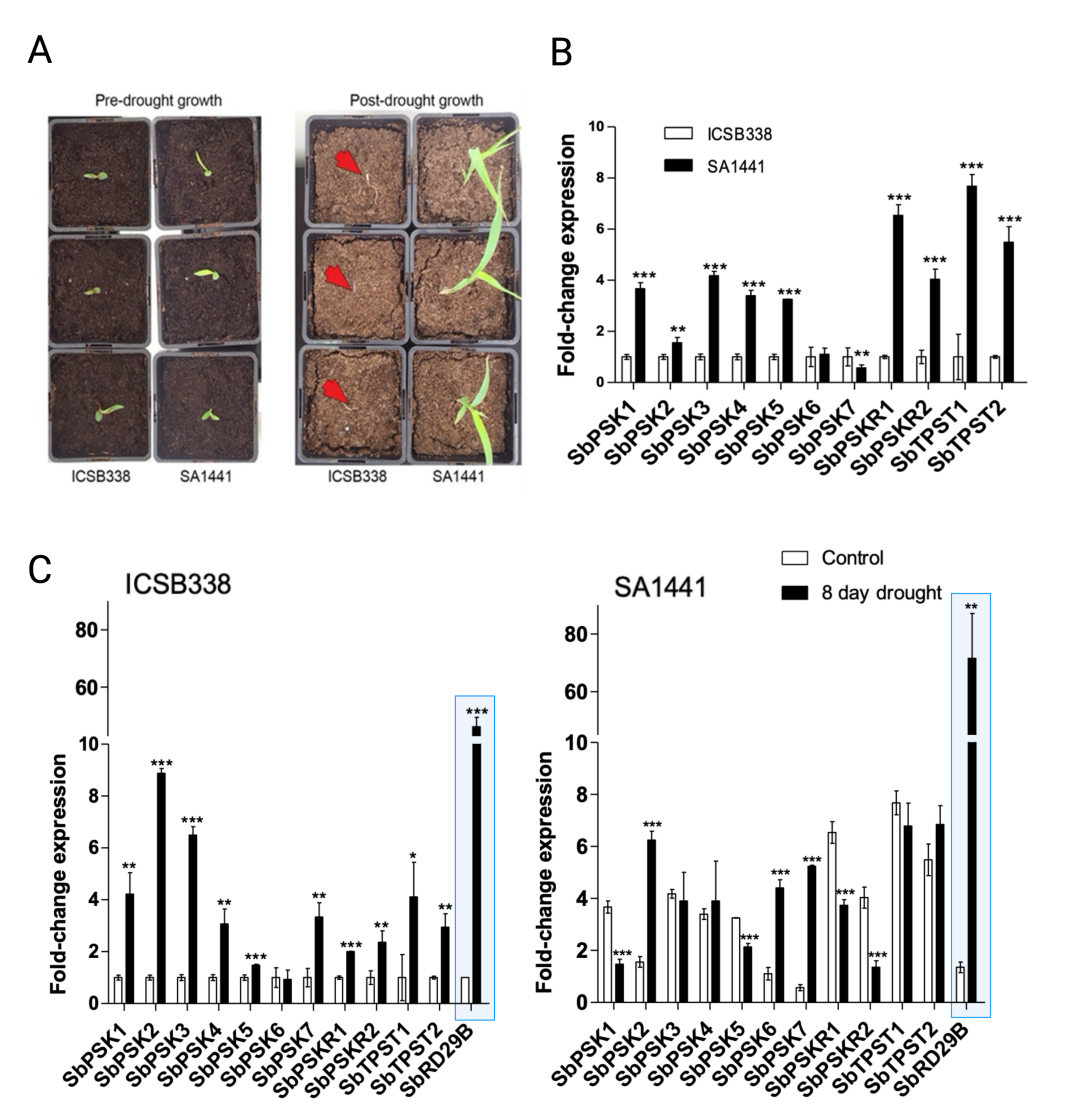
Expression of the *PSK* gene network and drought-marker gene in sorghum. **(A)** Four days after sowing, ICSB338 (drought-sensitive) and SA1441 (drought-tolerant) sorghum seedlings were transplanted into 22 cm-deep pots with 2.5 L fully hydrated soil. The seedlings did not receive any further water. Photographs were taken either 7 days (pre-drought growth) or 21 days (post-drought growth) after transplanting. Red arrows show the position of dead ICSB338 plants against the soil background. Drought-tolerant SA1441 plants continued to grow under limited water availability. **(B)** Sorghum plants were grown to the V3 growth stage (3 fully formed leaves with a fourth emerging) and either exposed to drought for 8 days or continued to receive water (controls). Root tissue was harvested for RNA extraction and qRT-PCR analysis of indicated target genes in samples from well-watered control plants. **(C)** Comparison of target gene expression between control and drought-treated plants in the two varieties. Bars represent means ± SD ( *n* = 3); single, double, or triple asterisks indicate significant differences between means at p less than or equal to 0.05, 0.01 or 0.001, respectively. Drought-marker gene highlighted in blue box.

## Description

Phytosulfokine (PSK) signalling has emerged as a crucial component of drought adaptation in investigated plants. PSK is a sulphated plant peptide that has been classified as a growth factor (Matsubayashi et al., 1997; Matsubayashi and Sakagami, 1996) . Recent research in Arabidopsis demonstrated that PSK activates specific signaling pathways and regulatory networks that confer drought resilience, although its functions extend to other stresses and reproductive processes (Takahashi and Shinozaki, 2019) . However, PSK research thus far has been limited to drought-sensitive species (Nagar et al., 2022; Reichardt et al., 2020; Stührwohldt et al., 2021) . Investigating the likely role of PSK signalling in a drought-tolerant crop like sorghum could provide fundamental knowledge of how drought tolerance works in nature. Use of sorghum as a model crop for drought stress tolerance has gained wider acceptance in this research field (Ngara and Ndimba, 2014) . Several groups have used sorghum as a drought-tolerant model using transcriptomics (Ananda et al., 2022; Dugas et al., 2011; Fracasso et al., 2016; Varoquaux et al., 2019) or proteomics (Goche et al., 2020; Ngara et al., 2018) , and physiological approaches (Li et al., 2020) .

We are studying plant adaptation to drought using two sorghum varieties with different response phenotypes: SA1441 is drought-tolerant while ICSB338 has considerable drought sensitivity ( Figure 1A ; Goche et al., 2020). In this study, we performed gene expression analysis of the PSK gene network in the two sorghum varieties under both optimum water availability and drought stress. Genes encoding tyrosylprotein sulfotransferases (TPSTs, which add a sulphate group to tyrosine residues of PSK peptide), PSK precursor peptides, and PSK receptors (PSKRs) were constitutively highly expressed in the drought-tolerant SA1441 sorghum not exposed to water deficit ( Figure 1B ). Only SbPSK6 and SbPSK7 genes were not expressed at a level significantly higher in SA1441 than ICSB338. Since there is gene redundancy in the PSK pre-peptides, the overall trend is that drought resistance is linked to constitutively high expression of PSK peptide genes. Nonetheless, SbPSK6 and SbPSK7 genes were subsequently upregulated on exposure to drought stress ( Figure 1C ). The drought-sensitive ICSB338 under optimum water conditions had significantly lower expression of the PSK gene network ( Figure 1B ). Notably, the drought-sensitive ICSB338 activated these genes only after exposure to drought stress, though SbPSK6 remained unresponsive ( Figure 1C ). The drought-sensitive ICSB338 response profile is like that observed in drought-sensitive Arabidopsis, where the PSK gene network was activated after drought perception (Stührwohldt et al., 2021) .

These findings implicate PSK signalling in natural drought tolerance of sorghum, and interestingly provides nuance to its deployment and potentially its role. Although PSK signalling is activated after perception of water deficits in ICSB338 sorghum ( Figure 1C ) and in Arabidopsis (Stührwohldt et al., 2021) , these plants remain drought-sensitive. However, PSK signalling is constitutively high prior to stress in SA1441 sorghum, and this is associated with remarkably superior drought tolerance ( Figure 1A ; Goche et al., 2020). While the PSK gene network does not respond to drought in SA1441 sorghum, the drought-marker gene SbRD29B remains inducible, indicating that no defects in inducible defences exist in this variety ( Figure 1C ). Our results implicate constitutive gene expression offering better stress protection than induced gene expression. Whether this is widespread in nature will be established by extending the study to more sorghum varieties and other drought-tolerant crops.

## Methods

SA1441 and ICSB338 seeds were sown in potted soil (216 cm3 by volume) and watered until they reached the V3 stage (3 fully formed leaves with a fourth emerging). While control plants continued to receive water as necessary, drought-treated plants were left without watering until harvesting the roots 8 days after the last watering. Three biological replicates were generated, each replicate consisting of root tissues pooled from three independent plants. RNA was extracted from the roots and analysed by quantitative reverse transcription-polymerase chain reaction as previously described (Goche et al., 2020) . Gene-specific primers are given in the reagents table. In sorghum, we identified seven SbPSK precursor genes, two genes encoding PSK receptor proteins (SbPSKR1 and SbPSKR2) and two tyrosylprotein sulfotransferase (SbTPST1 and SbTPST2) genes. The genes Sb04g003390 and Sb03g038910 were used as constitutive reference controls (Goche et al., 2020) . The sorghum RESPONSIVE TO DESICCATION 29B (SbRD29B) was used as a drought-marker. The REST2009 software version 2.0.13 was used for gene expression analysis. The Student’s t-test was used to compare gene expression at 5% confidence level using GraphPad Prism 5.00 software.

## Reagents

**Table:** 

**Name**	**Locus ID**	**Primer sequences**	**Function**
SbPSK1	Sobic.001G448300.1	TGCTGGCCATGAGAAGAGTG CGACCAACGTCCTCCTCATC	Target gene
SbPSK2	Sobic.008G034500.1	GAAGAAGAGCACATGGTGGTG GGACAGATAATACATAGCAGCAGTG	Target gene
SbPSK3	Sobic.008G034700.1	TGCGGCATCATCATCTCCTC CTGGGACTGAAGAAGGCAGG	Target gene
SbPSK4	Sobic.005G155200.1	TGCTGGCTACCTACATTCATGG GGCGTTGGTTTGGAGTTGAG	Target gene
SbPSK5	Sobic.002G021132.2	CCATTTCCACTCTCGCCCAC TTGCGCGTTTCTTGTTGGAG	Target gene
SbPSK6	Sobic.002G021132.1	GGTGGGTCGGTCGGTCAAG GGGGCCGGGTATTTGAAGGG	Target gene
SbPSK7	Sobic.005G035300.1	TCTCTGAGCACGCCTGAATG ACAGGGGTCACACACAGAAC	Target gene
SbPSKR1	Sobic.004G222100.1	TGAGCCCAAGGTTCGGTAAC AGAACTCCAACTTGCGGAGG	Target gene
SbPSKR2	Sobic.002G006100.1	GGACTTTGGGGTGTGGACAG ATGATGGAGAGTGGGAGGGG	Target gene
SbTPST1	Sobic.010G045966	AGCAATGCCCCGATTCCTAC AAGTTCTCTCCCTTGGCAGC	Target gene
SbTPST2	Sobic.010G045833	ACTGAGCCTCAAGGATTTGC AAACTGATGTCCAGTCCAGCG	Target gene
SbeIF4A1	Sb04g003390	GATGAGATGCTCTCCCGTGG TGATCTCTAGGGCCTCTGGG	Reference
Hypothetical	Sobic.03g038910	TCCTGAAGCATCTTTCCCTCC ACAGCCTGATTAGTTGGGGG	Reference
SbRD29B	Sobic.001G200700	GGGGAAGACGTGAAGGAAGG GGTATTCGTGTTCAGCTTCGC	Drought marker
